# Generation of Tβ4 knock-in Cashmere goat using CRISPR/Cas9

**DOI:** 10.7150/ijbs.34820

**Published:** 2019-07-03

**Authors:** Xiaocong Li, Fei Hao, Xiao Hu, Hui Wang, Bai Dai, Xiao Wang, Hao Liang, Ming Cang, Dongjun Liu

**Affiliations:** State Key Laboratory of Reproductive Regulation & Breeding of Grassland Livestock, Inner Mongolia University, Hohhot, 010000, China.

**Keywords:** CRISPR/Cas9, Tβ4, goat, knock-in, hair growth

## Abstract

The cashmere goat breed is known to provide excellent quality cashmere. Here, we attempted to breed high-yielding cashmere goats by specifically inserting the Tβ4 gene into the goat CCR5 locus and provided an animal model for future research. We successfully obtained Tβ4 knock-in goat without any screening and fluorescent markers using CRISPR/Cas9 technology. A series of experiments were performed to examine physical conditions and characteristics of the Tβ4 knock-in goat. The goat exhibited an increase in cashmere yield by 74.5% without affecting the fineness and quality. Additionally, RNA-seq analysis indicated that Tβ4 may promote hair growth by affecting processes such as vasoconstriction, angiogenesis, and vascular permeability around secondary hair follicles. Together, our study can significantly improve the breeding of cashmere goat and thereby increase economic efficiency.

## Introduction

The cashmere goat belongs to a fine breed that provides both cashmere and meat. Its main products cashmere, known as “fibre jewels” and “soft gold”, are sold all over the world. Particularly, Albas Cashmere Goat is known for the fibre fineness, which can be less than 14 microns, and meets the requirements of the international market. However, compared with other cashmere goats, the cashmere yield by this goat is low. Cashmere is derived from the hair follicles in the skin of cashmere goats, and hair follicles can be divided into primary hair follicles (PHF) and secondary hair follicles (SHF) depending on the size and shape. The fibres produced by PHF are coarse and hard, while those produced by SHF are thin and soft and are the main source of cashmere.

Thymosin beta 4 (Tβ4), a small molecule protein containing 43 amino acids, has many biological activities^1^. It is an important medium for cell migration and differentiation. Tβ4 may promote the migration of stem cells through its direct effect on cell migration or interaction with actin, and thereby promote hair growth. Tβ4 may also influence hair growth through matrix metalloproteinase-2 (MMP-2)^2^. In Tβ4 overexpressing mouse, it was found that Tβ4 promoted hair growth^3^. Therefore, we think that the Tβ4 gene knock-in at a safe site might improve production of cashmere.

A safe and reliable harbor site is needed for knocking in a foreign gene. “Safe harbor” can be defined by two criteria: the knocked-in exogenous gene should be stably expressed; and second, the expression of the endogenous genes around the integration site should not be affected^4^. Such “harbored” foreign genes can be expressed stably and efficiently, without deleterious effects on cells and tissues. In humans, only AAVS1 (adeno-associated virus integration site 1), CCR5 (chemokine receptor 5) and ROSA26 have been suggested as safe harbors^5^-^7^. Studies on AAVS1 and ROSA26 as safe harbors have been reported in mice and pigs^8^, ^9^. However, studies on CCR5 as a safe harbor are rare, especially in large animals and livestock. Nonetheless, studies have shown that individuals with congenital CCR5 deletion have no developmental or other abnormalities^10^, ^11^. Additionally, the site can stably express exogenous genes without affecting the expression of endogenous genes around the integration site^12^. Therefore, we selected CCR5 as a safe harbor in our study.

At present, fetal fibroblasts are mostly used as nuclear donor cells in somatic cell nuclear transfer (SCNT)^13^, ^14^. However, fetal fibroblasts allow only limited number of passages and are prone to aging. In our study, muscle-derived satellite cells (MDSCs)^15^ were used as nuclear donor cells. MDSC is a kind of adult stem cell with characteristics similar to embryonic stem cells; it has strong proliferative ability in vitro and is suitable for continuous passage and culture^16^. Furthermore, the presence of exogenous genes does not affect its stable passage, and there was no early apoptosis and tumorigenicity^17^.

CRISPR/Cas9 system has been widely used in gene editing due to its advantages of high efficiency, multiple target sites, short cycle, and easy screening^18^-^21^. We used the CRISPR/Cas9 system, with exogenous vectors to achieve integration of Tβ4 gene at a specific site. SCNT has wide application in construction of animal disease models, expanding propagation breeding stock, and protecting the endangered species^22^, ^23^. The combination of gene editing technology and SCNT overcomes the low efficiency and high cost of traditional methods to prepare gene editing large mammals, which is a great technological means for the cultivation of new species.

## Materials and Methods

### Ethics statement

We performed all experiments in compliance with the National Research Council Guide for the Care and Use of Laboratory Animals. All protocols were approved by the Institutional Animal Care and Use Committee of Inner Mongolia University. All animal experiments were accomplished at the Inner Mongolia YiWei White Cashmere Goat Limited Liability Company.

### Construction of the Cas9/gRNA expression vector

**Figure 1:**
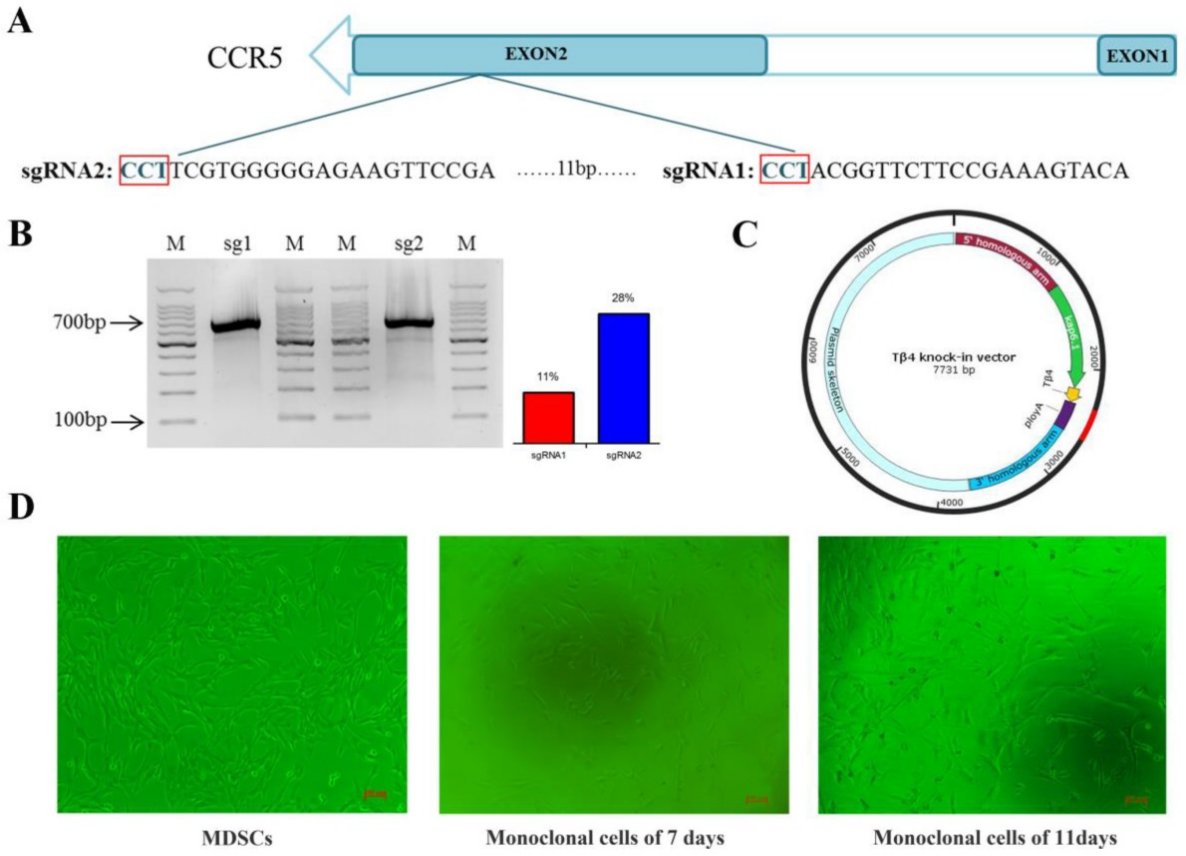
** CRISPR-Cas9 system mediates CCR5 gene targeting. A:** Schematic representation of sgRNAs targeting exon 2 of the CCR5 gene. **B:** The targeting efficiencies of sgRNA1 and sgRNA2 in MDSC were assessed by the Surveyor nuclease assay. “M” represents the marker, the efficiency of sgRNA1 is 11% and that of sgRNA2 is 28%. **C:** Plasmid map of Tβ4-knock-in vector. **D:** Cell line of MDSC, and different stages of monoclonal cells.

Two pairs of 20 bp target sequences (sgRNA1: CCTACGGTTCTTCCGAAAGTACA, sgRNA2: CCTTCGTGGGGGAGAAGTTCCGA) were designed on the second exon of the CCR5 gene (NC-022314.1) (Figure 1A) using CRISPR design tool (crispr.mit.edu). Cas9/gRNA expression vector was constructed according to the instructions of the Cas9/gRNA construction kit (Viewsolid Biotech, Beijing, China).

### Efficiency of Cas9/gRNA in MDSC

MDSC were cultured in Dulbecco's modified Eagle medium/F12 supplemented with 20% fetal bovine serum and 10% horse serum. The Cas9/gRNA plasmids (2 µg) were transfected via electroporation into MDSC with 225 V/2.5 ms according to the instructions of Nepa Gene (NEPA21 type II). After 48 h, DNA was extracted using the genomic DNA extraction kit (Promega, Madison, USA). The sequence containing the target site was amplified (Primer sequences are listed in Table S1) and the Surveyor Mutation Detection Kit (Transgenomic, Omaha, NE, USA) was used for evaluating the efficiency of sgRNA1 and sgRNA2.

### Construction of Tβ4 knock-in vector

The Tβ4 knock-in vector consisted of 5' homologous arm (1127 bp sequence upstream of target site), Kap6.1 promoter (1034 bp), the Tβ4 gene (135 bp), poly A sequence (270 bp), and a 3' homologous arm (1088 bp sequence downstream of target site) (Figure 1C). Keratin associated proteins (KAPs) are tissue-specific proteins composed of hair fiber matrix^24^. KAP6.1 is a member of the KAP family and has the characteristics of hair follicle specific promoter.

### Preparation of Tβ4 knock-in monoclonal cells

**Figure 2:**
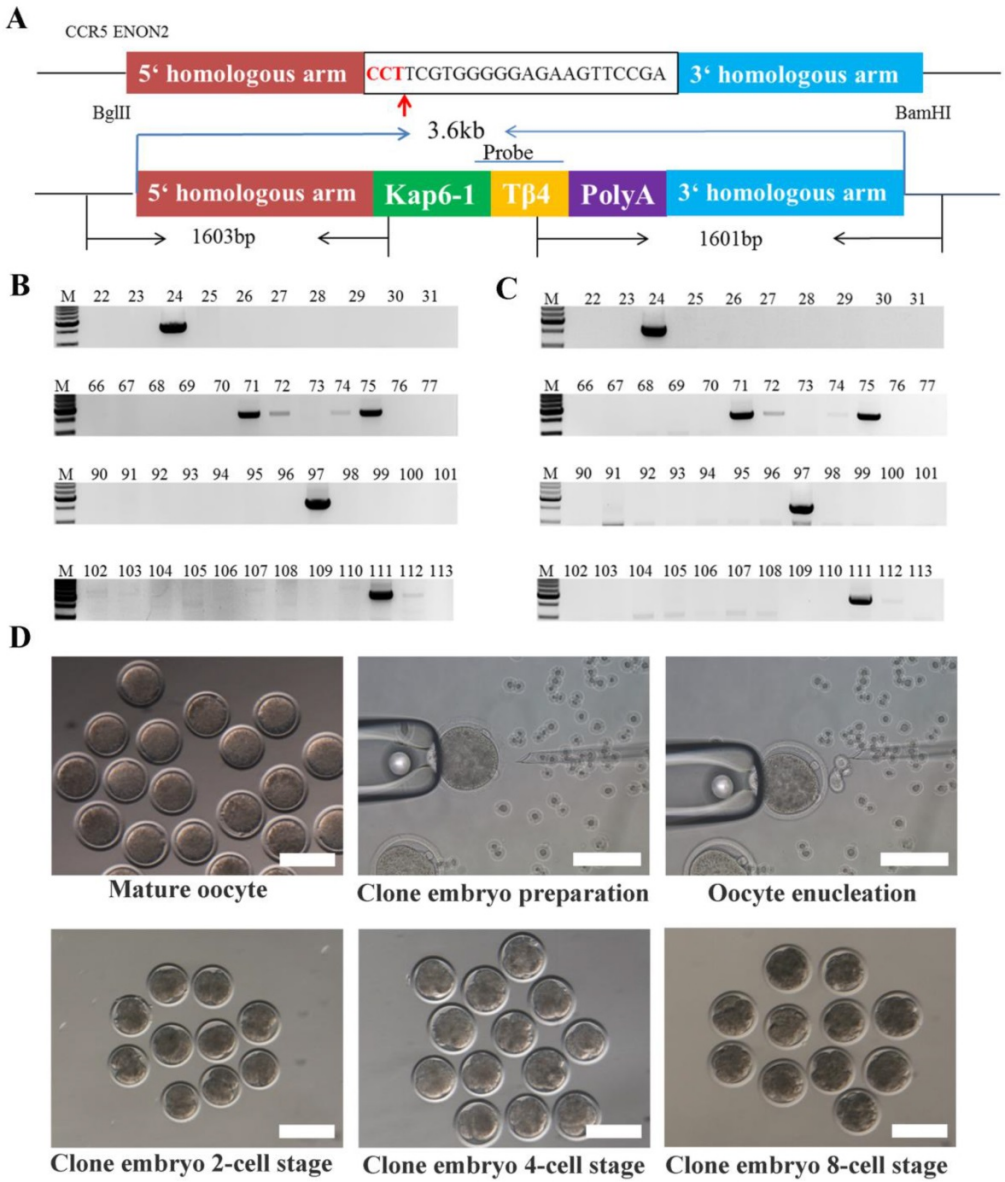
** Production of cloned embryos. A:** Schematic of CRISPR/Cas9 system-mediated gene knock in; two different primer pairs were designed to detect Tβ4 gene knock in, and the probe was used for Southern blot. **B:** Electrophoresis of PCR products of Tβ4-knock-in monoclonal cells amplified by 5'-junction primer. M. DNA marker; xx, number of clonal cell lines. **C:** PCR results of Tβ4-knock-in monoclonal cells amplified by 3'-junction primer. **D:** Cloned embryos at different developmental stages, Scale bar = 100 μm.

The Tβ4-knock-in vector (3 µg) digested with *Bgl*II and *Bam*HI, and the Cas9/gRNA plasmids (2 µg) were transfected into MDSC via electroporation. Single cells were inoculated into 96-well plate by flow cytometry (Figure 1D). To identify the Tβ4-knock-in monoclonal cells, we designed two pairs of PCR primers (Figure 2A, Table S2). The PCR conditions were 95°C for 5 min; 35 cycles of 95°C for 30 s, 58°C for 30 s, 72°C for 90 s; and 72°C for 10 min. The PCR products were detected by 1% agarose gel electrophoresis and sequenced. The positive monoclonal cell lines were used for SCNT.

### Generation of Tβ4 knock-in goat

The oocytes used in SCNT were collected from the local slaughterhouse and matured in vitro, and then enucleated. Next, a single donor cell was injected into the enucleated oocytes, forming the oocyte-donor cell complexes. The oocyte-donor cell complexes were fused by electric shock to form reconstructed embryos. After fusion, the reconstructed embryos were activated and cultured, and then cleavage was initiated. The cleavaged embryos with normal morphology were selected and transferred into the oviduct of oestrous goats.

### Identification of Tβ4 knock-in goat

Genomic PCR detection: To test whether Tβ4 gene was site-specific inserted, we performed two PCR reactions: the 5'-junction and the 3'-junction (Figure 2A, Table S2).

Southern blot: DIG High Prime DNA Labeling and Detection Starter Kit II (Roche, 11585614910) were used to detect the genomes of goats. The genomes were digested with *Bgl*II and *Bam*HI; the target fragments combined with probe was 3689 bp, and the length of probe was 527 bp (Figure 2A). Primers for amplification probe are shown in Table S3. The Tβ4 knock-in vector digested with *Bgl*II was used as the positive control.

Real-time PCR: mRNA was extracted from skin tissue of goats using RNAiso plus (TaKaRa Bio, Shiga, Japan). Real-Time PCR was performed on 7500 Real-Time PCR system. The expression of genes was analyzed by the method of 2^-ΔΔCt^. The levels of mRNA were normalized in relevance to GAPDH (Primer are listed in Table S4).

Immunocytochemistry: The fibroblasts were separated from ear tip tissues of goats and cultured. When the cells confluence reached 80%, they were fixed in 4% paraformaldehyde, and permeated with 0.5% triton X-100, blocked in 1% BSA. Primary antibodies against Tβ4 (ab14334) was diluted to 1:200 with 1% BSA, and incubated with samples overnight at 4°C. The secondary antibody (anti-rabbit IgG, Abcam) was diluted to 1:300 with 1% BSA and incubated with samples for 1 h at room temperature in dark. Then, samples were stained with DAPI and observed through confocal microscopy.

### Physical condition of Tβ4 knock-in goat

Off-target analysis: Potential off-target sites were predicted by freely available tool sgRNAcas9. We selected 9 potential off-target sites with high similarity to target sequences (Figure 4A). Genome of Tβ4-knock-in goat was used as template. PCR amplification was performed for sequences about 700 bp containing potential off-target sites (Table S7). The amplification products were sequenced.

Growth curve: The weight of the goats was measured every three months, from birth until the age of one year. A growth curve was drawn from five independent measurements.

Gene locus safety: The skin tissues of goats were used for mRNA extraction. Real-time PCR was used to detect the mRNA expression level of CCR5 gene and its adjacent genes, CCRL2, CCR2, and LTF. The levels of mRNA were normalized in relevance to GAPDH (Primers are listed in Table S4).

Blood physiological parameters: Blood was collected from the subjugular vein of goats and the blood tests were performed by the Veterinary Medical College of Inner Mongolia Agricultural University. The indices examined included counts of leukocytes, lymphocytes, monocytes, neutrophils, erythrocytes, platelets, and hemoglobin concentration.

### Characterization of Tβ4 knock-in goat

Paraffin section and hematoxylin and eosin (HE) staining: Skin tissues were taken from back of goats, fixed in 4% paraformaldehyde for 24 h, dehydrated with alcohol-xylene series, and embedded with molten paraffin. After solidification, the sample blocks were fixed on microtome and cut into 7-µm sections. After de-waxing, the sections were stained with HE to observe the morphology and number of hair follicles. In order to detect the ratio of SHF to PHF (S/P ratio), at least 10 skin tissue samples were analyzed.

Immunohistochemistry analyses: Tissue sections were de-waxed and heat-induced antigen retrieval was performed. Next, samples were blocked with 5% BSA. Primary antibodies against Tβ4 (ab14334) was diluted to 1:300 with PBS, and incubated with samples overnight at 4°C. Secondary antibody (anti-rabbit IgG, Abcam) was diluted to 1:1000 with PBS, and incubated with samples for 1 h at room temperature in dark. Then, samples were stained with DAPI and observed using confocal microscopy.

Quality of cashmere: Cashmere was collected from the back, side, shoulder, and thigh of goats before falling off. Collected samples were measured for strength extension, fineness, the quality ratio of cashmere to wool, and cashmere yield. We used YG006 electronic single fiber strength machine to measure the strength of cashmere. The results included the breaking force, breaking strength, and elongation at break. Optical diameter analyzer (OFDA2148) was used to measure the fineness of the cashmere. The quality ratio of cashmere to wool was measured by randomly selecting 2 g of samples, separating the cashmere and wool and evaluating their quality separately under the same temperature and humidity conditions. To measure yield, the cashmere were collected and weighted.

### RNA-seq analysis

Total RNA was extracted from skin tissues using TRIzol® reagent according to the manufacturer's instructions (Invitrogen). The RNA quality was determined by the 2100 Bioanalyser (Agilent) and quantified using the ND-2000 (NanoDrop Technologies). RNA sequencing was performed using HiSeq 4000 instrument (Illumina, San Diego, CA, USA). RNA Sequencing data statistics are shown in Table S8. Clean reads were separately aligned to the Capra hircus genome (NCBI:ID10731) with orientation mode using TopHat software^25^ (Tables S9,S10). To identify differentially expressed genes (DEGs) between different samples, the expression level of each transcript was calculated according to the fragments per kilobase of exon per million mapped reads (FRKM) method. RSEM 26 was used to quantify gene abundances. R statistical package software EdgeR (Empirical analysis of Digital Gene Expression in R) was utilized for differential expression analysis. GO functional enrichment and KEGG pathway analysis were carried out by Goatools and KOBAS 27.

## Results

### Efficiency of Cas9/gRNA mutation

The efficiencies of gRNA mutation were measured with the surveyor mutation test kit. Agarose gel (2%) electrophoresis was performed and the results showed that both sgRNA could mutate the genome at different degrees (Figure 1B). Gray level analysis was used to determine the mutation efficiencies of vectors Cas9/gRNA1 and Cas9/gRNA2 in cashmere goat muscle-derived satellite cells. The mutation efficiency of sgRNA1 was 11% and that of sgRNA2 was 28% (Figure 1B). Therefore, sgRNA2 was selected for subsequent experiments.

### Preparation of Tβ4 knock-in monoclonal cells

A total of 113 monoclonal cell lines were obtained, and subjected to PCR and sequenced. Among these, 7 monoclonal cell lines showed target bands in PCR detection (Figure 2B, Figure 2C), and were further sequenced, which showed correct sequences for all 7. Thus, the probability of obtaining positive monoclonal cell lines was 6.2%.

### Generation of Tβ4 knock-in goat by SCNT

**Figure 3:**
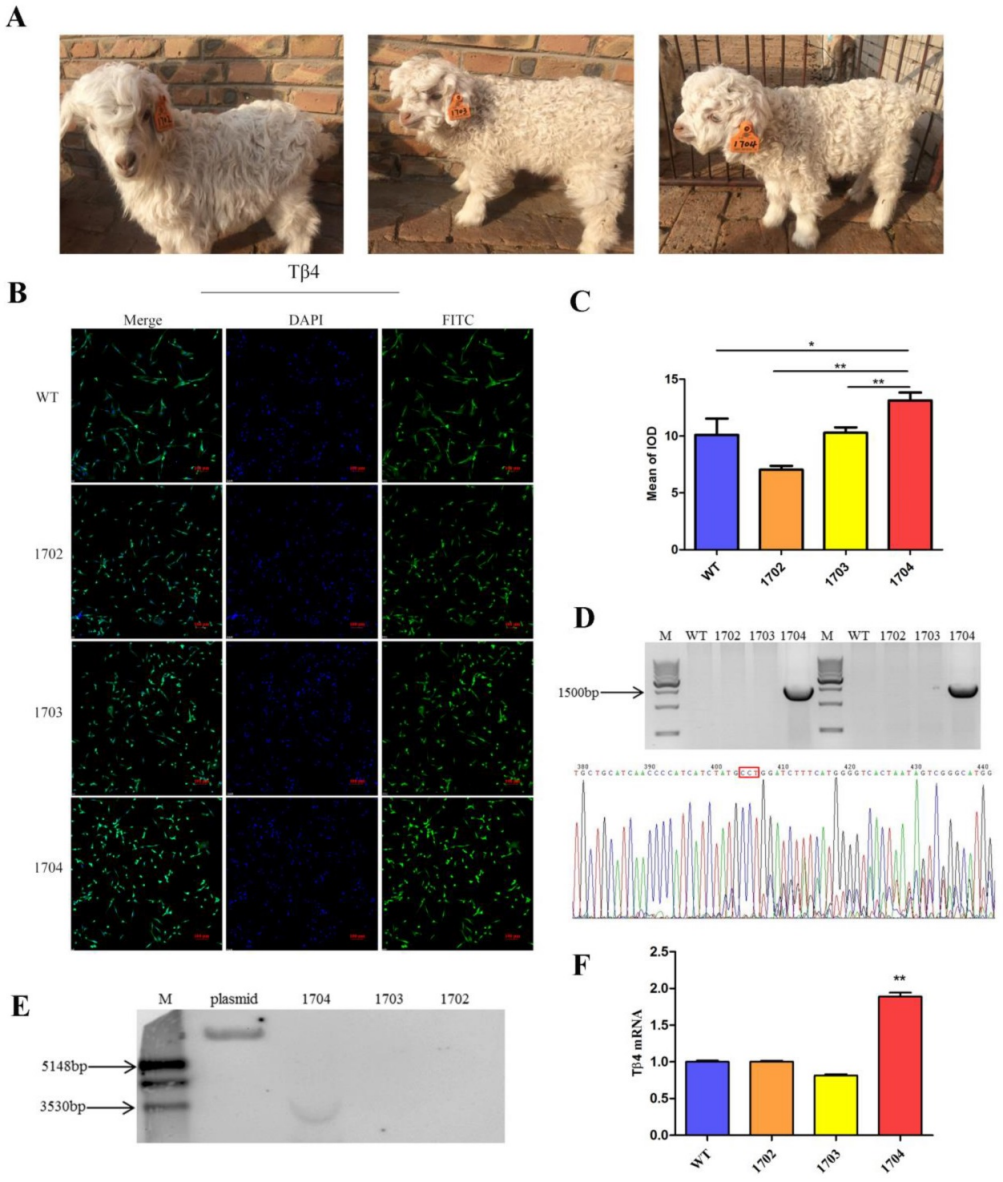
** Identification of Tβ4 knock-in goat. A:** Tβ4 gene knock-in goat (1704) and controls (1702 and 1703). **B:** Immunocytochemistry identification of the expression of Tβ4 gene in fibroblasts separated from the WT, 1702, 1703, and1704 goats. **C:** Quantitative analysis of fluorescence signals by Image pro plus. **D:** PCR detection by two pairs of primers showed in Fig. 2A and sequenced results of the integration sites. **E:** Southern blot confirmed site-specific insertion; target fragments combined with probes is 3.6kp. **F:** Real-time PCR identification of the Tβ4 gene in WT, 1702, 1703 and1704 goats. Error bars represent mean ± SD. * *p* < 0.05; **p < 0.01.

The Tβ4 knock-in monoclonal cell line was used as nuclear donors for SCNT. A total of 738 oocytes were collected from 174 ovaries, of which 447 oocytes were matured, with the maturity rate being 60.6%. Microscopic manipulation resulted in 439 reconstructed embryos, of which 314 were successfully fused by electric shock, with a fusion rate of 71.5%. The fused reconstructed embryos were activated and cultured in vitro till cleavage. A total of 176 reconstructed embryos showed cleavage, with a cleavage rate of 56% (Table S5). Once the embryos developed into 2 cells, 4 cells and 8 cells (Figure 2D), they were transplanted into 56 oestrus receptors of ewes. Finally, one Tβ4 knock-in goat, numbered 1704, was successfully bred. (Figure 3A, Table S6).

Meanwhile, we used untreated MDSC as control for SCNT. Of the 38 transplanted recipient ewes, two goats were successfully bred, numbered 1702 and 1703 respectively (Figure 3A, Table S6).

### Identification of Tβ4 knock-in goat

Wild Type (WT), 1702, and 1703 were used as controls. WT obtained by normal fertilization. 1702 and 1703 obtained by SCNT with untreated MDSC as nuclear donors. We used two pairs of primers to identify whether the Tβ4 gene is site-specific integration. (Table S2). Only the 1704 goat presented expected bands (Figure 3D), which were then sequenced. The results showed that the 1704 goat achieved integration of Tβ4 gene at the CCR5 locus. The results were further verified by Southern blot analysis, which showed probe binding at 3689 bp in the 1704 genome (Figure 3E). Real-time PCR analysis also showed that the mRNA expression of Tβ4 gene was increased in 1704 goat (Figure 3F). The results of immunohistochemistry showed that the immunofluorescence intensity of 1704 goat was stronger than controls (Figure 3B). Image pro plus was used to quantify the fluorescence signal (Figure 3C). Together, our results indicated that the 1704 goat had achieved integration of Tβ4 gene specifically at the CCR5 site.

### Physical condition of Tβ4 knock-in goat

**Figure 4:**
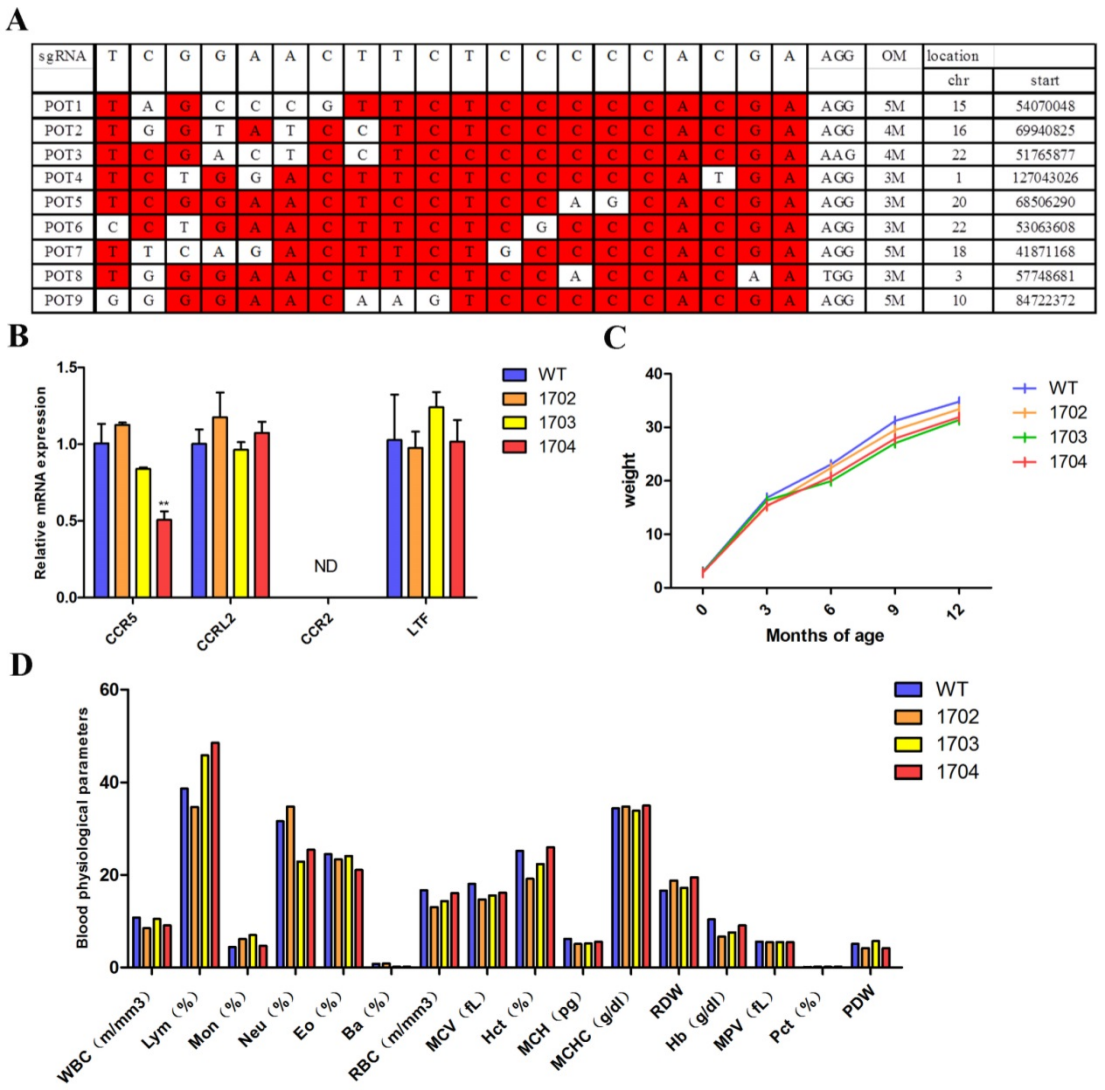
** Physical condition of Tβ4 knock-in goat. A:** 9 potential off-target sites predicted by sgRNAcas9. **B:** Real-time PCR was performed to analyze the expression of CCR5 gene and its adjacent genes in WT, 1702, 1703, and 1704 goats. Error bars represent mean ± SD. * *p* < 0.05; **p < 0.01. **C:** Growth curve based on weight of the cashmere goats measured at different periods. **D:** Blood physiological parameters of four goats.

Although the CRISPR/Cas9 system has great advantages in gene editing, it can show off-target phenomenon^28^, ^29^. In order to test off-target mutations, we performed amplification, sequenced, and analysis of 9 potential off-target sites. The results showed that there was no off-target mutation. Although no off-target was detected in the 9 potential off-target sites, it is uncertain whether they occurred at undetected sites. Upon monitoring the growth curve of the four goats, it was found that there was no significant difference in growth status between the 1704 goat and controls (Figure 4C). Blood routine test showed no significant difference in the indexes of the four goats (Figure 4D). Genes adjacent to CCR5 were examined by real-time PCR, and no significant differences were found (Figure 4B). The above results showed that 1704 goat displayed no off-target effects in our examination. Further, the body indexes of 1704 were normal and the genes adjacent to the CCR5 gene were not affected.

### Characterization of Tβ4 knock-in goat

**Figure 5:**
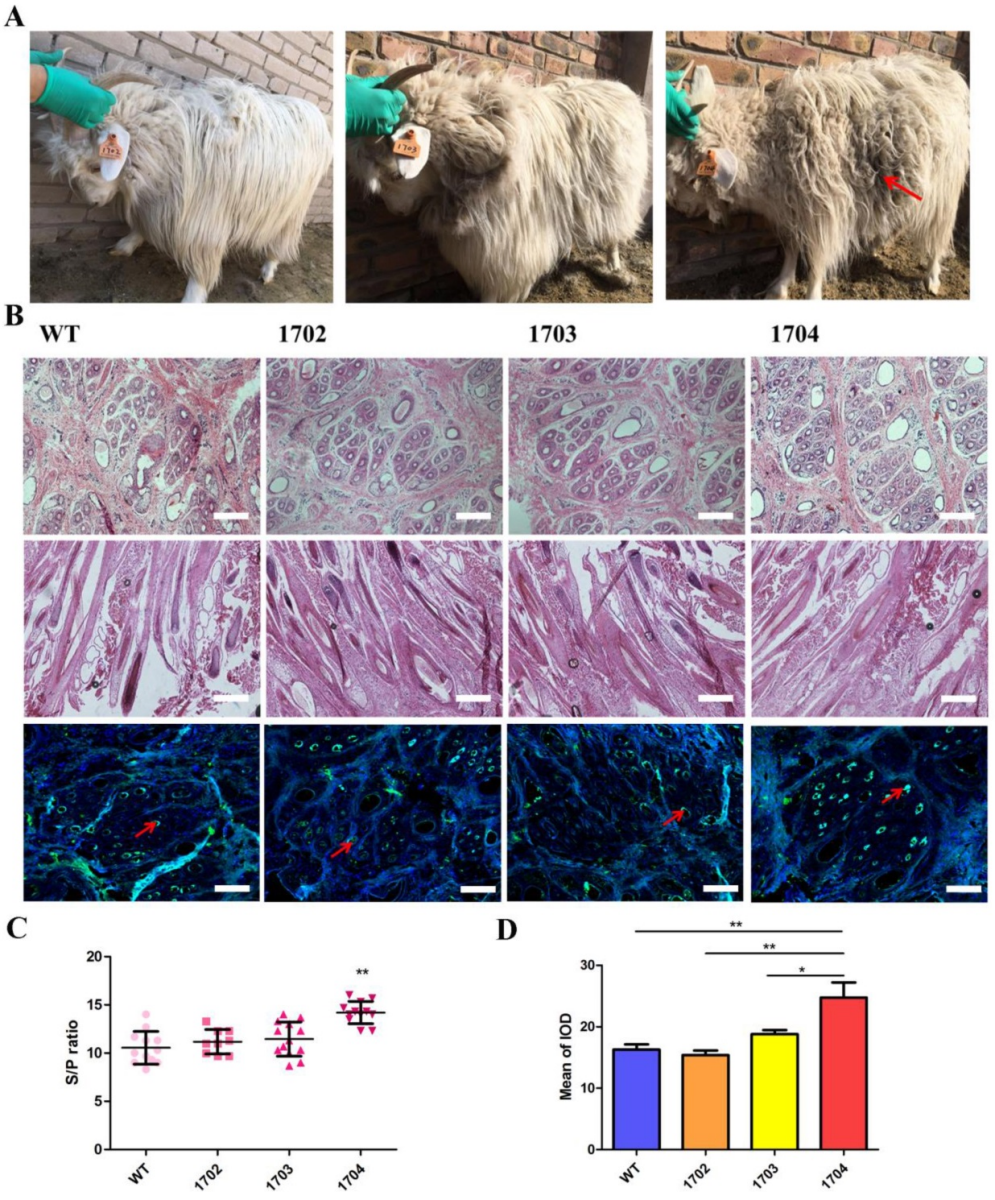
** Characterization of Tβ4 knock-in goat. A:** One year old Tβ4 gene knock-in goat (1704) and controls (1702 and 1703). **B:** Cross-sectional and longitudinal-section images of skin tissues from WT, 1702, 1703 and 1704 goats, and immunohistochemical detection of Tβ4 in hair follicle tissues. Scale bar = 100 μm. **C:** The S/P ratio of WT, 1702, 1703 and 1704 goats. Error bars represent mean ± SD. * *p* < 0.05; **p < 0.01. **D:** Image pro plus was used to quantify the fluorescence signal of immunohistochemical. Error bars represent mean ± SD. * *p* < 0.05; **p < 0.01.

**Figure 6:**
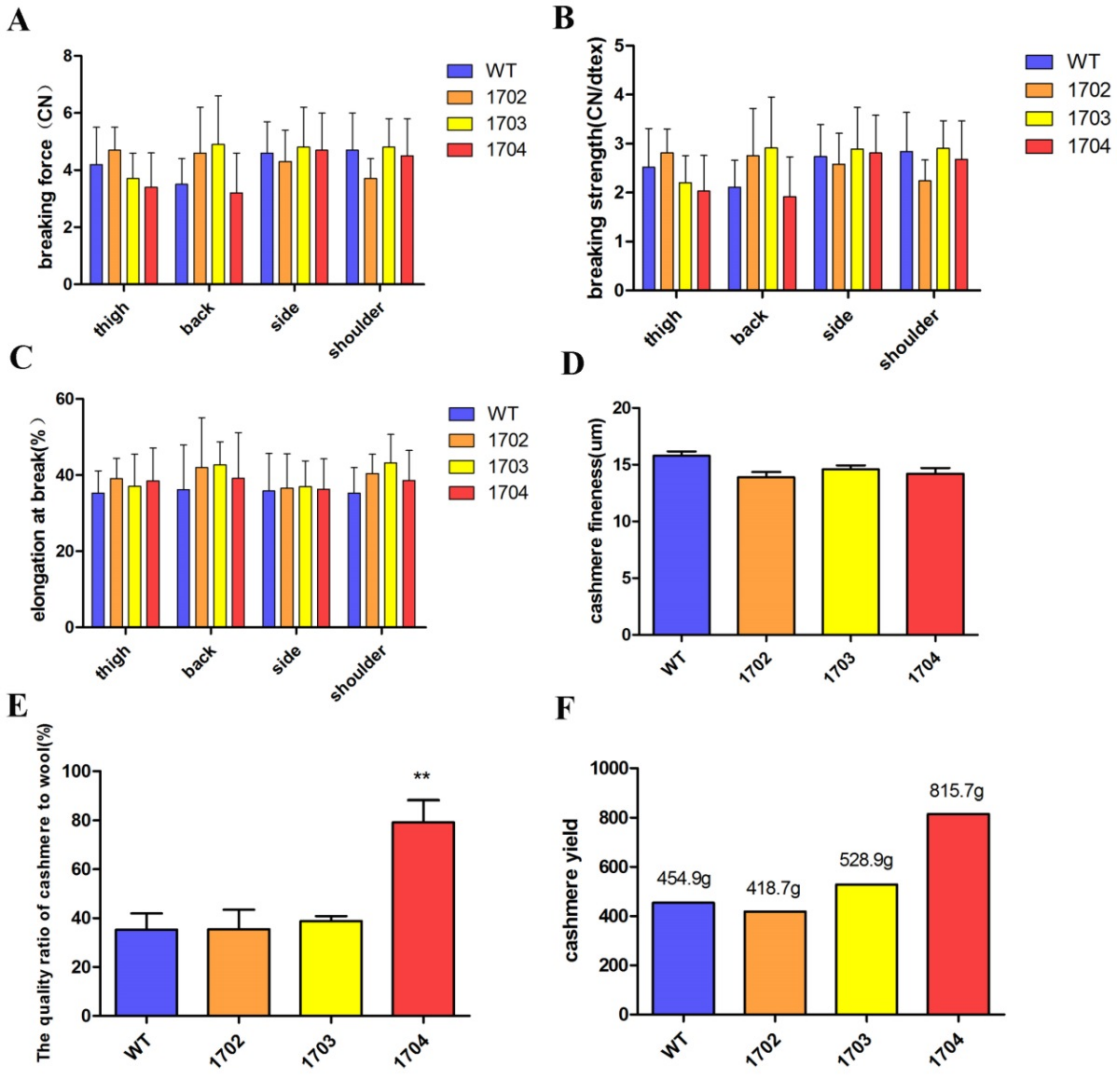
** Quality of cashmere. A:** Breaking force of cashmere obtained from different parts of the four goats. **B:** Breaking strength of cashmere from different part of four goats. **C:** Breaking elongation of cashmere from different part of four goats. **D:** The fineness of cashmere obtained from the four goats. **E:** The quality ratio of cashmere to wool obtained from the four goats. Error bars represent mean ± SD. * *p* < 0.05; **p < 0.01. F: The cashmere yield of four goats.

Paraffin sections and HE staining were performed on the back skin of four cashmere goats (Figure 5B). The results showed that the S/P of 1704 goat was 14.2 (Figure 5C), which was 28.4% higher than controls. Longitudinal section showed that the follicle structure of 1704 goat was complete (Figure 5B). Immunohistochemistry showed that Tβ4 gene was mainly expressed in secondary follicles, and the expression of Tβ4 gene in secondary follicles of 1704 goat was higher than that in controls (Figure 5B). Image pro plus was used to quantify the fluorescence signal (Figure 5D).We also detected the strength extension (Figure 6A-6C), fineness (Figure 6D), the quality ratio of cashmere to wool and the cashmere yield. The results showed that the quality ratio of cashmere to wool of 1704 goat was 79.2% (Figure 6E), which was significantly higher than controls. The cashmere yield of 1704 goat was 815.7 g (Figure 6F), which was increased by 74.5% when compared with controls. From the appearance, the phenotype of 1704 goat had changed obviously (Figure 5A). The strength extension and fineness showed no significant difference. These results indicate that Tβ4 gene mainly acts on SHF, increasing the number of SHF to increase cashmere yield. Additionally, the cashmere yield was increased without affecting the cashmere quality.

### RNA-seq analysis

**Figure 7:**
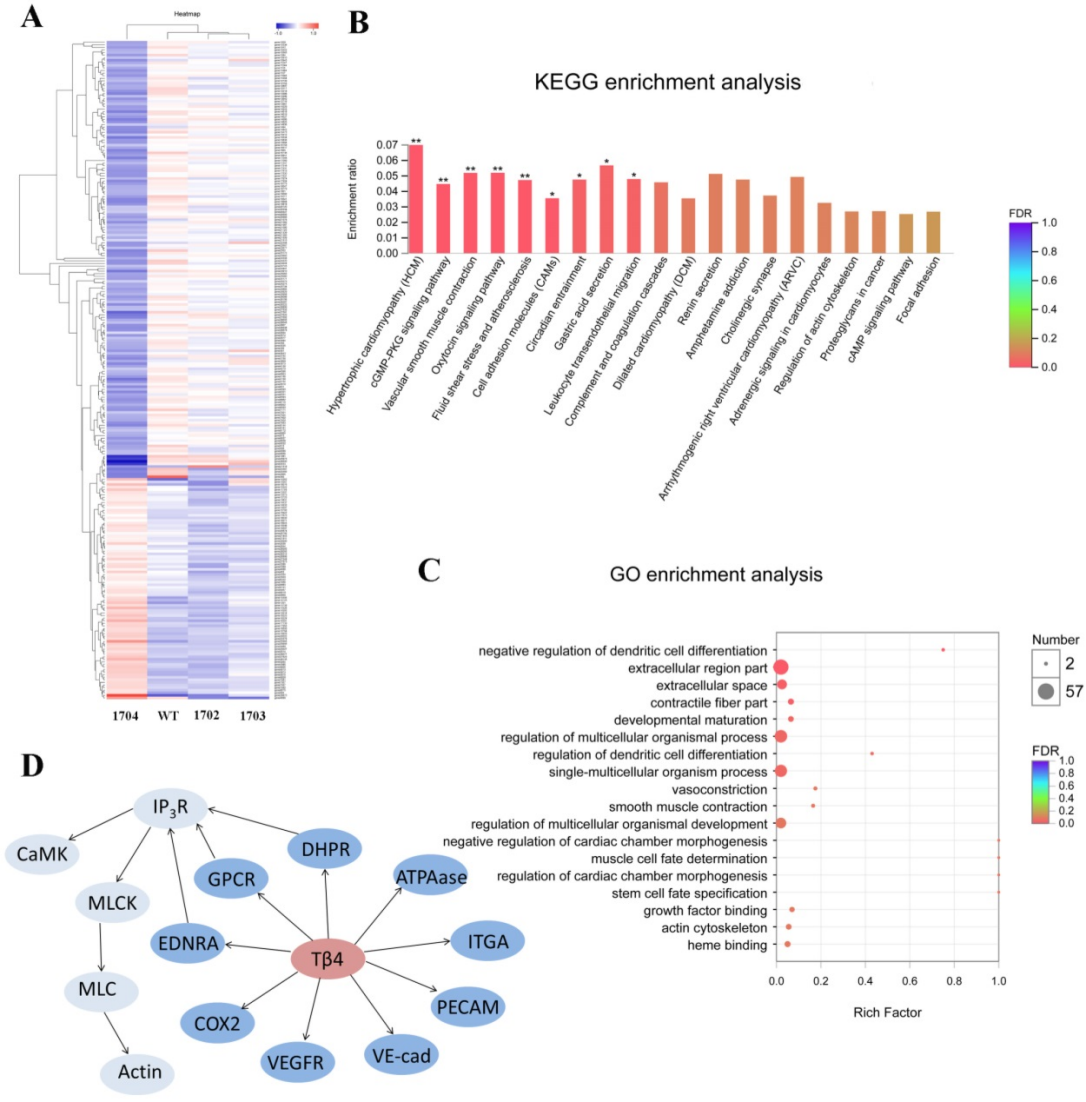
** RNA-seq analysis. A:** Heatmap of differentially expressed genes. Red indicates higher expression genes in the sample, and blue indicates lower expression. **B:** KEGG enrichment analysis of differentially expressed genes. **C:** GO enrichment analysis of differentially expressed genes. **D:** Differentially expressed genes induced by Tβ4 gene knock-in, and interaction of these genes in the highly enriched pathway.

To explore the effects of Tβ4 gene, we compared transcripts of 1704 goat and controls. P-value < 0.05 and |fold change (FC)| > 1 were set as the thresholds for identifying DEG. Notably, a total of 256 genes were found to be significantly altered in the 1704 goat. Of these, 86 genes were increased, whereas 170 genes were decreased in the 1704 goat (Figure 7A). The subsequent Gene Ontology (GO) analysis showed these significantly altered genes were associated with the biological processes of regulation of dendritic cell differentiation, vasoconstriction and smooth muscle contraction (Figure 7C). Further pathway analysis of the DEGs revealed that oxytocin signaling pathway, vascular smooth muscle contraction signaling pathway, cGMP-PKG signaling pathway, and fluid shear stress and atherosclerosis signaling pathway were highly enriched (Figure 7B). The DEGs associated with these enriched pathways mainly included membrane proteins, such as vascular endothelial cytokine receptors (VEGFR), platelet endothelial cell adhesion molecule (PECAM-1), and vascular endothelial cadherin (VE-cad); and genes regulating contraction of smooth muscle, such as inositol triphosphate receptor (IP3R), calmodulin (CaM), myosin light-chain kinase (MLCK), and myosin light chain (MLC) (Figure 7D). These findings indicate that Tβ4 affects a series of processes related to blood vessels.

## Discussion

Most of the genes editing animals are traditionally prepared by randomly integrating a sequence of genes into the genome. However, the integration site and copy number of exogenous genes directly affect the expression of exogenous genes^30^, ^31^. Usually, only a small number of genetically edited animals show some excellent desired traits and are stably inherited to the next generation. Although gene targeting technology, developed by the principle of DNA homologous recombination^32^, ^33^, allows precise modification of the genome, the technique is extremely inefficient, which limits its application. In contrast, the CRISPR/Cas9 technology has the characteristics of high efficiency, multiple target sites, short cycle and easy screening, which provides great advantages for site-specific genomic modification.

With the development of gene editing technology, the safety of gene editing animals has been of great concern. The function and safety of the target gene, the major components of the expression vector, the integration sites of the target gene and the off-target phenomenon in the CRISPR/Cas9 system all affect the safety of gene editing animals. In the current study, the target gene we selected, Tβ4, is an endogenous gene of the cashmere goat, and many studies have shown that Tβ4 can promote hair growth. The main components of the expression vector constructed in our experiment include homologous arm sequence, promoter sequence, target gene sequence and a polyA sequence, without any addition of fluorescent or screening markers. In addition, the follicular specific promoter KAP6.1 was used to express Tβ4 only in follicle cells to improve the production of cashmere, and prevent the expression of Tβ4 in other tissues. By examining off-target effects, growth monitoring, routine blood and mRNA expression levels of CCR5 adjacent genes, we demonstrated that the gene editing goat was largely safe.

The strength extension of cashmere mainly determines the fastness and abrasive resistance of wool fabrics and quality of related products. Fineness is an important indicator to determine the quality and value of cashmere. The quality ratio of cashmere to wool can be used to measure the production potential of the cashmere goat. A series of examination indicators in our research showed that integration of Tβ4 gene at specific sites could improve the cashmere yield without affecting the fineness and quality of cashmere, which is of great significance for the breeding of high-yielding cashmere goats.

In our study, the DEGs we screened out such as VEGFR, PECAM-1, and VE-cad, play important roles in promoting vascular endothelial cell division, angiogenesis, leukocyte migration, changes in vascular permeability, and maintenance of vascular integrity^34^-^38^. The DEGs IP3R, CaM, MLCK, and MLC together regulate smooth muscle contraction and relaxation processes^39^-^41^. Thus, we found that the Tβ4-mediated promotion of hair growth may be related to a series of processes such as vasoconstriction, angiogenesis, and vascular permeability changes around secondary hair follicles. Future studies will focus on elucidating the mechanism of Tβ4 gene promoting hair growth. The Tβ4 knock-in goat we obtained in this study will be a good animal model for future research.

In conclusion, we used CRISPR/Cas9 technology combined with SCNT technique and MDSC as nuclear donors obtained cashmere goat that integrated Tβ4 genes at the CCR5 locus. The Tβ4 knock-in goat showed excellent characteristics, with increased cashmere yield by 74.5% compared with controls without affecting the fineness and quality of cashmere. Our results also revealed that the Tβ4-mediated promotion of hair growth may be related to a series of processes such as vasoconstriction, angiogenesis and vascular permeability changes.

## Supplementary Material

Supplementary figures and tables.
