# Supplementary material for: Generation of Tβ4 knock-in Cashmere goat using CRISPR/Cas9
Source: Int J Biol Sci. 2019 Jul 3;15(8):1743–54. doi: 10.7150/ijbs.34820 (PMC6643211; doi:10.7150/ijbs.34820)
Supplement: Supplementary file 1 — Supplementary figures and tables. [file ijbsv15p1743s1.pdf]

Supplementary material

Table S1. Primer of sgRNA efficiency detection

name	Primer sequences（5'-3'）	Product size
F	GTGGTGGCTATGTTTGCCTCTC	714bp
R	AGAGCTGATGCCAAATTGATGGATG	

Table S2. Primers of positive cell detection

name	Primer sequences（5’-3’）	product size
5'-juntion-F	ACGTGGCTACACAACATCCAA	1603bp
5'-juntion-R	GCTAAACCAAGTCCCTTATCAACA	
3'-juntion-F	GGAAACGCAAGAGAAAAATCCACT	1601bp
3'-juntion-R	TAGGGTGGTGTTCAGCTTACAA	

Table S3. Primer of probe amplfication

name	Primers sequences	Product size
Southern-F	5’ -AACAAACTGAGGTCAAGAGCAA-3’	527bp
southern-R	5’ -CCTGCTTGCTTCTCCTGTTCA-3’	

Table S4. qPCR primers

name	Primers sequences(5'-3')
GAPDH-F	CGTGTCCGTTGTGGATCTGA
GAPDH-R	GAGTGTCGCTGTTGAAGTCG
Tβ4-F	AAACCCGATATGGCTGAGATTG
Tβ4-R	GCCTGCTTGCTTCTCCTGTT
CCR5-F	TCAAACATCAACTCCCCTCTATGAC
CCR5-R	GCCCACAAAACCAAAGATGAA
CCRL2-F	CAAAGGACTCAAGCAAGCAGAA
CCRL2-R	CTATGGAGAAGAGCATCAGGAGAA
CCR2-F	AACCCTGCCGAAAGACCA
CCR2-R	GCCCACAAAACCAAAGATGAA
LTF-F	CGTGAAGAAGGGCAGCAAC
LTF-R	CATCAACGCAGGGAACACA

Table S5. Preparation of clonal embryos with Tβ4 gene knock-in

Type	Oocytes	Mature oocytes	Cloned embryos	Fused mbryos	Activated embryos	Embry type		
						2-cell	4-cell	8-cell
Tβ4 knockin	738	447	439	314	176	67	86	23
Negative cell	489	314	269	231	167	63	80	24

Table S6. The birth of Cashmere goats

Recipient goat number	Date of lambing	Donor cell source	Goat number	Lamb sex	Birth weight of lamb
308265	26-Feb-17	Normal fertilization	WT	♀	3.02kg
106085	27-Feb-17	Negative monoclonal cells	1702	♀	2.85kg
008225	2-Mar-17	Negative monoclonal cells	1703	♀	2.93kg
917208	5-Mar-17	Positive monoclonal cells	1704	♀	2.82kg

Table S7. Primers for amplifying predicted off-target sites.

Primers	Sequences(5'-3')	Product size(bp)
POT1	GAGTTGCCCATGTTCCCTGTT	470
	CAGGGCTTGTCTTGTCATCCT	
POT2	GTGACTAAGCCCACACACAGAG	704
	TCAGTCGTGTCCAGCTCTTTG	
POT3	TGACTGCCGTAAGCATAGACC	582
	CCACGGGAAAAGGGCTATGA	
POT4	TCACGGCTCCTTAGAGTGGA	817
	GGTCACTAAGAGTCGGGCAC	
POT5	GCAAAGCAGAATGCAAGGACA	721
	CCCTTCATTTCCACAGCGAAG	
POT6	ACAAACCCTGGCGGCTTATT	604
	ATTCAGGTTACGCCTGTGCC	
POT7	TGCAGCTCAAGCGGTAAAGA	652
	CTCCACTGAGGTAACAGGCG	
POT8	TTGGGAAACACTGCTGTAAACC	689
	TCTTATTCCAGTCTCAAGTAGGC	
POT9	ACCACAGTTTGCTGTCCTCC	701
	CGCGGTACTGGAGTGTAATCA	

Table S8. RNA Sequencing data statistics

Sample	Raw reads	Raw bases	Clean reads	Clean bases	Error rate (%)	Q20 (%)	Q30 (%)	GC content (%)
WT	51736378	7812193078	51434892	7700137954	0.023	98.87	96.1	53.14
1702	54363166	8208838066	53932144	8064104295	0.023	98.88	96.17	53.76
1703	53345278	8055136978	52945120	7918150722	0.023	98.86	96.11	53.13
1704	51287570	7744423070	50948896	7622637982	0.0233	98.77	95.84	52.63

Table S9. RNA sequencing Comparison statistics

Sample	Total reads	Total mapped	Multiple mapped	Uniquely mapped
WT	51434892	50194656(97.59%)	2749820(5.35%)	47444836(92.24%)
1702	53932144	52553210(97.44%)	3373753(6.26%)	49179457(91.19%)
1703	52945120	51509678(97.29%)	3323618(6.28%)	48186060(91.01%)
1704	50948896	49562391(97.28%)	5141933(10.09%)	44420458(87.19%)

Table S10. Different regions of reads distribution

Sample	Introns	3'UTR	CDS	5'UTR	Intergenic
WT	3495445.0 (4.48%)	12336409.0 (15.8%)	58188114.0 (74.51%)	2564497.0 (3.28%)	1507845.0 (1.93%)
1702	6640943.0 (8.87%)	11004141.0 (14.7%)	53037313.0 (70.84%)	2559642.0 (3.42%)	1628699.0 (2.18%)
1703	3712394.0 (4.79%)	12411064.0 (16.03%)	57196908.0 (73.85%)	2541162.0 (3.28%)	1585632.0 (2.05%)
1704	7104742.0 (10.1%)	11053338.0 (15.71%)	47763026.0 (67.9%)	1997868.0 (2.84%)	2421111.0 (3.44%)

DNA seqence results of 5'-junction:

GATGCTCTCCGTCTAGTAACAGAGTGTTAGTCGCTCAGGCGTGTCTGACTCTTATG
ACCCCATGGATTGTAGCCCACCAGGCTCCACTGACCATGGAATTCTCCAGGCAAG
AATACTGGAGTGGGTTGCCATTTCCTTCTCCAGGGGATCTTCCCAACCCAGGGATT
GAATCCAGGTCTCCTGCATTGCAGGCAGATTCTTTACCATCTGAGCCACCACGGAA
ACCCAAAGCAGAGAAGCTAGCAGCAAACTAATATAAAAAGTTCACTGTTTGACAA
AAAAAAGGACTTCAGTTAAATGTAGAAATCTATGTATCAATTTTTAAAACCTACTTA
AGTATATAAAACGGTTTGCATTCATGATGGACTGCTAAGGACATTCTAGGACTTTAT
AAAACACCTTTTCTTTATTTACAGAGTCAAGCAAAATGGATTATCAAACATCAACT
CCCCTCTATGACATTGATTATGGGATGTCAGAGCCATGCCAAAAAATCAACGTGAG

GCAAATTGCAGGCCAGCTCTTGCCCCCACTCTACTCGCTGGTGTTCATCTTTGGTTT
TGTGGGCAACTTGCTGGTTGTCCTCATCCTGATAAACTGCAAAAAGCTGAAGAGC
ATGACTGACATCTATCTGCTCAACTTGGCCATCTCTGACCTACTTTTCATCATCACTA
TCCCATTCTGGGCTCACTACGCTGCAGACCAGTGGGTATTTGGAAATACAATGTGC
CAGTTATTCACAGGGTTCTATTTCATTGGTTATTTTGGTGGAATCTTCTTCATCATCC
TCTTGACAATCGATAGGTACCTGGCTATCGTTCATGCTGTGTTTGCTTTAAAAGCCA
GAACAGTCACCTTTGGGGCAGTGACAAGTGGGGTCACGTGGGTGGTGGCTATGTT
TGCCTCTCTCCCAGGAATTATCTTTACCAAATCCCAAAAGGAAGGCTCTCGTCATA
CGTGCAGCCCACATTTCCCATCCAATCAGTATCATTTCTGGAAGAGTTTCCAAACTT
TAAAGATAGTCATCTTGGGGCTGGTGCTGCCTCTGCTTGTCATGATCGTCTGCTACT
CGGGAATCATAAAAACCCTGCTCCAGTGTCGCAGCGAGAAGAAGAAGCACAAGG
CTGTGAGGCTCATCTTCGTGATCATGATTGTCTACTTTCTCTTCTGGGCTCCCTACA
ACATCGTCCTCCTCCTGAGCACCTTCCAGGAATTCTTCGGCTTGAATAACTGCAGT
GACTCTAACAGGCTGGACCAAGCCATGCAGGTGACAGAGACCCTGGGGATGACG
CACTGCTGCATCAACCCCATCATCTATGCCTGGATCTTTCATGGGGTCACTAAGAGT
CGGGCATGGCTGAGCGACTTCACTTTCATGTATCACTTTCATGCATTGGAGAAGGA
AATGGCAACGCACTCCAGTGTTCTTGCCTGGAGAATCCCAGGGCTGGGGGAGCCT
GGTGCACTGCCATCTCTGGGGTCGCACAGAGTCGGACATG

DNA seqence results of 3'-junction:
TCGGGGGAGGCTAACTGAAACACGGAAGGAGACAATACCGGAAGGAACCCGCGC
TATGACGGCAATAAAAAGACAGAATAAAACGCACGGTGTTGGGTCGTTTGTTCATA
AACGCGGGGTTCGGTCCCAGGGCTGGCACTCTGTCGATACCCCACCGAGACCCCA
TTGGGGCCAATACGCCCGCGTTTCTTCCTTTTCCCCACCCCACCCCCCAAGTTCGG
GTGAAGGCCCAGGGCTCGCAGCCAACGTCGGGACGGCAGGCCCTGCCATAGGTC
GACAACTATCTCCTACGGTTCTTCCGAAAGTACATCGCCAGCCGCTTCTGCAAAGG
CTGTCAGTCTTCCAGGGAGAGGCTCCAGAGCGAGTGAGCTCCGTTTACACACGAT
CCACGGGAGAACAGGAAGTCTCTGTTGGCTTGTGATCTGACTCAGTTCATATATGC
AAACTGTGGGGGAGCAGTTCAAGAGGAAATTACTGTCAACAAGGGTTTAAGATTC
ATCCATCAATTTGGCATCAGCTCTAAATATATTAGATATTTCAAGCCCATCAATTCTA
GAAAGCCAAAGCAAAACACGCTGATGAAATAGCAATCTTCTCACCGCCCCCCTCC
ACATACAACAATTTATTGGCAAGCTCTCCCCTCACTACAAAAGGTTCAATGTTTAA
AAAAAAAAATCCTCAGAGAATTATTAATTCCTGAGTTTGGTTACCTGAACAGGAAT
AACAAAATGAACTGAGGAAAGTATTGTATAGTTTCTTATCTGGGTAGGGCAATAGC
CAGGTTGCAAATGTGATTAAAATAGGTCCTTCTCTTGCCATGGGGAGAAAAGACAT
GCCGGTGATCAGATAAGGAATGACATCTTCCATGTGGGATCTCTCCCAAAAGGTAC
GTTAATAAGTTCCACAGACACTGATGCCAAGGAAGAGCCCTGTGGTCTGCTGAGA
GCTGGGAAGGCTTCTTCGCAGAAAAGGTACTGGAGGCCAATGGTCTGTCAGTGGA
GAAGGAAGCTGAGCTCCAGGATGCAGGCACTGCACAGGCAAAACTTGGCTGTGG
GGAGACAGGCACTGGCTGGGGGAGCTCCTGGGAGGAAAAATGAGGCTGGTGCAT
GAGAAAACTGGACGGCATTGCTCATCAAATTCAGAGAGCAGAGTGGGGAGCCCT
GGCCAGTGTTGCAGAAAGCTCATTCTGTAACCAAAGGATGGCCTGGAAAGGTGAG
CATTCAGGTCAAGGAGACCAGCAACAATGTGATCAAGTGAGGAGGCTCCACTAAA

GTTGAAGCCAGAGATGGGAAGGATGGATACCACCTCACAGCACTGAGGATGAGAG
CCAGCAGAATTTGGGGTGGATTTGGCTTGGCAGTGAAGGGCAGAGAGGAATCAG
AGACTCCCTAGATTTGAGGCAAGCATCAGAGGTGCCCTAAAAGAGACATCAAGCA
TGGAAGGAGGAGGAGGTTTAGGTCAAA
